# Genetic analysis of the *FOXL2* gene using quantitative real-time PCR in Chinese patients with blepharophimosis-ptosis-epicanthus inversus syndrome

**Published:** 2011-02-09

**Authors:** Shanshan Hu, Junjing Guo, Binbin Wang, Jing Wang, Zhou Zhou, Guangkai Zhou, Xuchen Ding, Xu Ma, Yanhua Qi

**Affiliations:** 1 Department of Ophthalmology, the 2nd Affiliated Hospital of Harbin Medical University, Harbin, China; 2 National Research Institute for Family Planning, Beijing, China

## Abstract

**Purpose:**

The purpose of this study was to identify the mutation(s) or deletion(s) of the forkhead box protein L2 (*FOXL2*) gene in Chinese patients with blepharophimosis-ptosis-epicanthus inversus syndrome (BPES).

**Methods:**

Genomic DNA extracted from peripheral blood was collected from two Chinese families and from one sporadic case. PCR direct sequencing and quantitative real-time PCR-based copy number screening for the whole exon of *FOXL2* were performed.

**Results:**

Direct sequencing revealed an indel mutation c.50C→TA in the sporadic case which resulted in a frameshift generating 78 novel amino acids and terminating prematurely at codon 95. Deletions in the *FOXL2* gene were confirmed by quantitative real-time PCR (q-real-time PCR) in two families in which intragenic mutations were excluded by direct sequencing. These changes containing deletions and a de novo mutation were not detected either in the non-carrier relatives or in 100 normal controls.

**Conclusions:**

This study identified two deletions and a de novo mutation in the *FOXL2* gene in Chinese BPES patients. This is the first study to report *FOXL2* gene deletions detected by q-real-time PCR in this ethnic group. This technique enriches the diagnostic methods of molecular genetics in BPES patients. The de novo mutation expands the mutation spectrum of *FOXL2*.

## Introduction

Blepharophimosis-ptosis-epicanthus inversus syndrome (BPES, OMIM 110100) is a rare genetic disorder characterized by eyelid malformation and ovarian dysfunction. Based on the presence or absence of premature ovarian failure (POF), two clinical types have been distinguished: type I is associated with POF in affected females, whereas type II is not [1]. BPES is primarily inherited in an autosomal dominant manner, but may also occur sporadically, although the autosomal recessive pattern has also been reported in one consanguineous family [2]. According to cytogenetic rearrangements [3] and linkage analyses [4-6], BPES had been mapped to the human chromosome 3q23 region. Subsequently, the *FOXL2* (forkhead box protein L2, OMIM 605597) gene was identified as the pathogenic gene for BPES [7]. Furthermore, non-syndrome POF and granular cell tumors of the ovary may be associated with *FOXL2* mutations [8,9].

**Figure 1:**
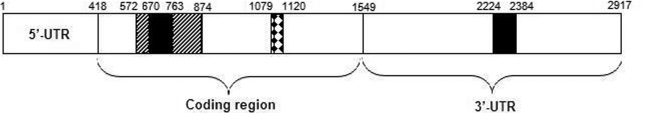
Diagram of the *FOXL2* gene and positions of the amplification segments by the q-real-time PCR primers in *FOXL2*. Shaded areas represent the DNA-binding domain and the polyalanine tract in the coding region of *FOXL2*, respectively. Black boxes indicate the location of the amplification segments of two pairs of primers with respect to the nucleotide (top) numbering.

The FOXL2 protein comprising 376 amino acids is a member of the large family of winged-helix/forkhead transcription factors that play important roles in a variety of developmental processes [10]. FOXL2 contains an unique DNA-binding domain of 100 residues with amino acid positions from 52 to 152, and a polyalanine tract of 14 residues with amino acid positions from 221 to 234 (Figure 1). A comparative analysis shows that the entire open reading frame (ORF) of *FOXL2* is highly conserved in several vertebrate species [11]. Expression studies have shown that the FOXL2 protein is expressed in the mesenchyme of developing mouse eyelids and in fetal and adult ovarian granulosa cells, which is consistent with the preconceived role of *FOXL2* in early eyelid development and ovarian maintenance [7].

To date, more than 125 mutations have been reported in individuals with BPES type I and II. Among all genetic defects found in BPES, an estimated 72% of cases are due to intragenic FOXL2 mutations [12]; 2% of cases involve cytogenetic rearrangements containing unbalanced translocations and interstitial deletions of 3q23 [13]; about 12% of BPES cases result from deletions involving partial or whole *FOXL2* gene deletion and contiguous gene deletion, including *FOXL2* and adjacent gene(s) [13]; and about 5% of cases involve regulatory deletions outside the *FOXL2* gene [14]. Using multiplex ligation-dependent probe amplification (MPLA) and quantitative PCR [15], deletions leading to *FOXL2* haploinsufficiency may be detected in individuals with typical BPES in which intragenic mutations were excluded by sequencing of the *FOXL2* ORF.

The purpose of this study was to identify the mutation(s) or deletion(s) of *FOXL2* in two Chinese families and one sporadic case with BPES, using the technique of PCR direct sequencing and quantitative real-time PCR (q-real-time PCR). This is the first report deletion detection in the *FOXL2* region using q-real-time PCR in a Chinese ethnic population.

## Methods

### Patients

Five BPES patients and four of their relatives were recruited from Heilongjiang province in the northeast of China, as well as two unrelated families (F1, F2) and a sporadic case (S1). Informed consent was obtained from their parents or guardians. One hundred healthy normal controls were also involved in this study. The study protocol conformed to the ethical guidelines of the 1975 Declaration of Helsinki and was approved by the Heilongjiang Institutional Review Board (Harbin, China).

Clinical information about the patients was obtained from an ophthalmologist, an endocrinologist, and a gynecologist using the standardized diagnostic criteria. Where possible, facial photographs were obtained with patient approval.

### Mutation detection

Blood samples from BPES patients and their healthy relatives were collected and stored at −20 °C. Genomic DNA was extracted from peripheral blood leucocytes using a QIAamp DNA Mini kit (Qiagen, Hilden, Germany).

The whole exon of the *FOXL2* gene containing coding and flanking regions was amplified by the polymerase chain reaction (PCR) method using the primers listed in Table 1. The PCR reaction mixture (50 µl) contained 12.5 µl 2× GC Buffer II, 200 µmol/l dNTP mix, 1 unit of LA Taq polymerase (TakaRa Biotechnology Co. Ltd., Dalian, China), 10 pmol primer pairs and 100 ng genomic DNA. PCR cycling conditions were as follows: pre-degeneration at 95 °C for 3 min, then 38 cycles of denaturation, annealing and extension, followed by a final extension at 72 °C for 10 min. The PCR products were directly sequenced on an automated sequencer (ABI 3730XL Genetic Analyzer; Applied Biosystems, Foster City, CA) to perform mutation analysis.

**Table 1:** List of the primers and annealing temperatures used for the amplification of the whole exon of the *FOXL2* gene containing coding and flanking regions with BPES.

**Primer**	**Sequence (5′-3′)**	**Product length (bp)**	**Annealing temperature (°C)**
AF	GTGGAGCCCATACGAATCAG	610	62
AR	GTACGAGTACGGGGGCTTCT		
BF	CAGCGCCTGGAGCGGAGAG	545	64
BR	CTTGCCGGGCTGGAAGTGC		
CF	GACCCGGCCTGCGAAGACA	517	66
CR	GGCCGCGTGCAGATGGTGT		
DF	CGCGGCCGCTGTGGTCAAG	500	68
DR	GCTGGCGGCGGCGTCGTC		
EF	CCTCTTTGTCCCCTCAGTTTA	467	51
ER	CGGTGTAAACCGAGTACAGG		
FF	AGAAAGGGACGGACCAATAC	518	55
FR	CAGATAGGGAGAGGGTGAAAC		
GF	GAAGTATTGTGGCCTTGGAGT	542	55
GR	ATTTATTCGGGAATCGACAAG		

### Quantitative real-time PCR analysis

The real-time primers, as listed in Table 2, were designed and provided by TakaRa (TakaRa Biotechnology Co. Ltd.). The amplification segments of the two pairs of primers were located in the 5′ and 3′ ends of the *FOXL2* gene, respectively (Figure 1). The gDNA was used as template in q-real-time PCR reactions with SYBR^®^ Green PCR Master Mix (TakaRa Biotechnology Co. Ltd.) and performed using a 7000 real-time PCR system (Applied Biosystems). The quantification of the target sequences was normalized to an assay of chromosome 21, C2, and the relative copy number (RCN) was determined on the basis of the comparative ΔΔcycle threshold (Ct) method with a normal control DNA as the calibration standard [16]. The experiments were repeated three times. A ≈0.5-fold change in RCN was used as the benchmark for deletion.

**Table 2:** List of q-real-time PCR primers.

		Position in chromosome 3*	
**Primer**	**Sequence (5′→3′)**	**Start**	**End**
foxl2–1F	CATCGCGAAGTTCCCGTTCTA	140147983	140148003
foxl2–1R	CACCTTGATGAAGCACTCGTTGA	140147910	140147932
foxl2–2F	GCTATCAGTCCCGTCGCTTC	140146430	140146449
foxl2–2R	TTAGCAAACTCCAAGGCCACA	140146289	140146309
C2-F	CAATTCAGGTCAGGTGATAACTCAGTAA	-	-
C2-R	GCCAGGTTTAGAATGTTTGTCTAAGTC	-	-

*The position of the primers are given with respect to the Human Genome Browser Gateway.

## Results

### Clinical findings

**Figure 2:**
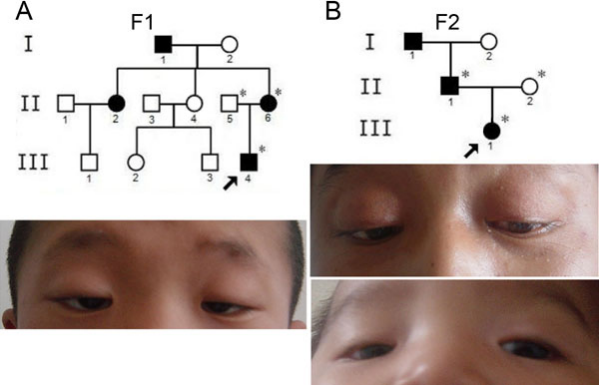
Eyelid photographs and pedigrees of BPES patients from two families. **A**: Pedigree of family 1 (F1) and picture of eyelid feature of the proband. **B**: Pedigree and photographs of eyelid feature observe of the proband and father in family 2 (F2). Squares circles indicate males and females, respectively, and the black symbols represent patients. Asterisks indicate analyzed individuals. The arrows indicate the proband.

All three probands indicated typical features of BPES, including small palpebral fissure, ptosis of the eyelids, epicanthus inversus, and telecanthus. In addition to eyelid malformations, other eye abnormalities were detected in some of these patients. In family 1 (Figure 2A), the proband (F1-III:4) was diagnosed with bilateral amblyopia and strabismus. His mother (F1-II:6), who had undergone eyelid surgeries in childhood, did not suffer from POF at the time of this study, and was diagnosed BPES type II. In family 2 (Figure 2B), the BPES type of the female child (F2-III:1) could not be determined. Her father (F2-II:1) was a 27-year-old BPES patient who presented some abnormalities such as bilateral amblyopia, strabismus, and ophthalmoplegia, besides ocular abnormalities characteristic of BPES. In addition to the typical ocular manifestations of BPES, the 3-year-old girl in the sporadic case (S1) had no other developmental abnormalities. Neither of her parents had clinical evidence of this disorder. Clinical data for the patients in this study are summarized in Table 3.

**Table 3:** The clinical findings of patients in this study.

**Individual**		**Type of BPES**	**Clinical data**
F1*	II:6	II	32-year-old female with refractive error and normal levels of sex hormones.
	III:4		7-year-old boy with bilateral amblyopia and strabismus, no clinical data about mental retardation.
F2*	II:1	Undetermined	27-year-old male with bilateral amblyopia and strabismus and ophthalmoplegia.
	III:1		1-year-old girl with the normal growth development and the undetermined BPES type.
S*	S1	II	3-year-old girl with the risk of having POF, no clinical data about other abnormal development.

*F1=family 1; F2=family 2; S=sporadic case.

### Mutations in *FOXL2*

**Figure 3:**
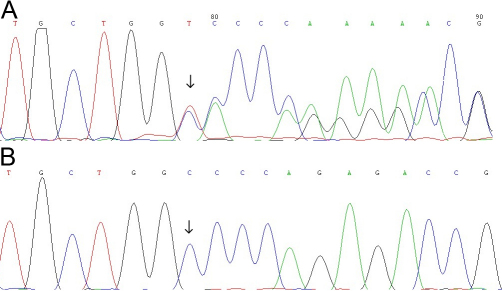
Mutation analysis from sporadic case (S1). **A**: Partial nucleotide sequences of *FOXL2* from patient. The sequence showed an indel mutation, c.50delCinsTA (indicated by the arrow). This de novo mutation resulted in a frameshift mutation expected to cause miscoding of 78 amino acids from codon 17 and eventually a premature stop codon at 95. **B**: The parents and the control subjects lacked this change.

In the sporadic case (S1), bidirectional sequencing of the whole exon of *FOXL2* revealed an indel mutation, C→TA at nucleotide 50 (c.50 delCinsTA, Figure 3A). This was a frameshift mutation expected to cause miscoding of 78 amino acids from codon 17, and eventually a premature stop codon at codon 95. This change was not detected in either of proband’s parents or in the 100 normal controls (Figure 3B).

### Quantitative real-time PCR analysis

**Figure 4:**
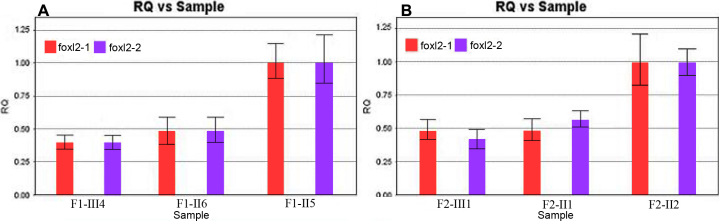
Quantitative real-time PCR analysis from two families. Deletions of CNVs in *FOXL2* are shown in BPES patients from family 1 (**A**) and family 2 (**B**), respectively. The histogram of amplification products of the two pairs of q-real-time PCR primers indicate that the mean gene copy number in samples from BPES-affected family members was half that of samples from unaffected family members.

Since intragenic mutations were excluded by direct sequencing of *FOXL2* in the two BPSE families, we then considered whether *FOXL2* gene deletion existed in the patients. This was subsequently confirmed by the q-real-time PCR technique. The study found that the relative copy numbers (RCNs) of the patients were about half that of the healthy individuals (Figure 4). Copy number variations (CNVs) measured by q-real-time PCR signified that the deletion of *FOXL2* led to haploinsufficiency. To confirm the CNVs found in the present study, the q-real-time PCRs were performed at least three times to eliminate handling error. As expected, no deletion was detected either in non-carrier relatives or in BPES-free controls.

## Discussion

In two families (F1, F2) with BPES, two deletions in *FOXL2* were detected and characterized by an efficient technique using q-real-time PCR. Compared to fluorescent in situ hybridization (FISH) and multiplex ligation-dependent probe amplification (MLPA) analysis, q-real-time PCR appeared to be more convenient [15]. In the present study, the copy numbers of the amplification segments located at the 5′ and 3′ ends of *FOXL2* were approximately 50% that of healthy individuals (Figure 4), which suggested that *FOXL2* was deleted in the region encompassing these amplification segments. Since *FOXL2* deletions are found in at least 12% of BPSE cases [13], deletion screening is now routinely used for molecular diagnosis of BPES. Based on the predictions of Beysen et al. [14], deletions encompassing *FOXL2* have no reliable genotype-phenotype correlations with regard to POF. However, according to a study by D’haene et al. [15], *FOXL2* deletions may be associated with varying degrees of ovarian dysfunction. Therefore, we attempted to assess the BPES type in the two families. In family 1, the absence of female infertility or POF in II:6 suggested that this gene deletion did not affect the ovarian expression of *FOXL2*, and thus led to BPES type II. However, in family 2, the BPES type could not be determined owing to the prepubertal developmental stage of the female child. *FOXL2* deletions causing haploinsufficiency of this gene may affect ovarian function, leading to POF with a variable age of onset [15]. Therefore, apart from the ophthalmological follow-up, young female patients of undetermined phenotype require a close endocrinological and gynecological follow-up. Importantly, this study is the first to report a *FOXL2* gene deletion in a Chinese ethnic population detected by quantitative real-time PCR. Consequently, we have identified q-real-time PCR as a relatively reliable, convenient and inexpensive molecular diagnostic tool for deletion screening of *FOXL2*, which will facilitate genetic counseling for BPES patients and help identify those female patients who require an extended clinical follow-up for POF.

BPES features typically include epicanthus inversus (fold curving in the mediolateral direction, inferior to the inner canthus), low nasal bridge, and ptosis of the eyelids leading to both vertical and horizontal narrowing of the palpebral fissures. Thus, subjects with BPES have smaller than normal eyelid openings. The ptosis is usually bilateral and symmetric. Additional dysmorphic features of the eye include nystagmus, microphthalmos, microcornea, and stenosis of the lateral canaliculi [17]. The F1 and F2 families shared similar features to those described above that characterize BPES, including ptosis of the eyelids, epicanthus inversus and telecanthus. Other ocular abnormalities in the F1 and F2 families included bilateral amblyopia, strabismus, and ophthalmoplegia, which may not be common features of BPES. Apart from the eye abnormalities, patients carrying deletions in *FOXL2* presented more frequently associated clinical findings. D'haene et al. [15] commented that psychomotor retardation was noted in some patients with a haploinsufficiency of the *FOXL2* gene. Microcephaly was reported in some cases with large deletions of *FOXL2* involving the neighboring ataxia telangiectasia and Rad3 related (*ATR*) gene [18]. As reported here, however, III:4 in F1 carrying a deletion in the *FOXL2* region presented normal psychomotor and mental developments at age 7 years, which suggested that there was not an exact genotype-phenotype correlations attributed to deletions of the *FOXL2* region. Owing to the infancy of III:1 in F2, associated clinical findings such as psychomotor delay and microcephaly could not be determined. Therefore, it may be important for providing a prognosis regarding associated clinical findings in new borns with BPES carrying a *FOXL2* deletion.

In a 3-year-old girl with sporadic BPES (S1), we found a de novo mutation which had a C deletion associated with a TA dinucleotide insert at position 50, resulting in a frameshift generating 78 novel amino acids and terminating prematurely at codon 95. The mutation led to a truncated protein in which the entire forkhead DNA-binding domain was erased; this was not found in her parents who lacked clinical evidence of the disorder. Predictions from De Baere et al. [19] suggested that intragenic mutations that resulted in proteins truncated before the polyalanine tract probably led to BPES type I. Therefore, the female child will need regular evaluation by an endocrinologist or gynecologist to explore the possibility of sterility or to anticipate POF [20].

The deletions of *FOXL2* reported in this study which caused the haploinsufficiency leading to the presence of a null allele may result in a disable transcript undergoing nonsense-mediated decay [21] as a causative mechanism for BPES. It has been reported that the truncated protein formed as a result of intragenic mutations is strongly aggregated in the nucleus [11]. Aggregation of the protein seriously impairs its DNA-binding function, and then influences interactions with the other proteins. Since the entire polyalanine tract of the COOH-terminus of FOXL2 is important for transcriptional repression of the steroidogenic acute regulatory (*StAR*) gene [22], either the *FOXL2* deletion or mutation-dependent protein truncation before the polyalanine tract might increase *StAR* expression, thereby resulting in the development of POF. The clinical findings and genetic analysis in the sporadic case, together with findings that intragenic mutations can seriously impair the function of the FOXL2 protein [11] suggest that a single mutation in FOXL2 can cause complete inactivation of the gene product. As a result, the genetic mutation in the sporadic case can, in effect, produce the same disruption in *FOXL2* gene function as gene deletions, leading to development of the BPES and POF phenotypes.

In conclusion, this study provides the first report of *FOXL2* gene deletions in a Chinese ethnic population detected by quantitative real-time PCR. It supports the application of quantitative real-time PCR techniques as a relatively reliable, convenient and inexpensive method for detecting genetics abnormalities in BPES patients. Meanwhile, the de novo mutation in the sporadic case broadens the mutation spectrum of *FOXL2*. The new information concerning mutations in *FOXL2* and the more widespread use of q-real-time PCR for deletion screening is likely to facilitate the clinical genetic diagnosis of BPES and lead to improved genetic counseling for a larger number of BPES patients.
